# Tool-HLCA: An intervention to promote the health literacy of school-aged children

**DOI:** 10.1093/eurpub/ckac129.247

**Published:** 2022-10-25

**Authors:** T Schulenkorf, U Bauer, O Okan

**Affiliations:** 1 Department of Sport and Health Science, Technical University Munich, Munich, Germany; 2 Faculty of Educational Science, Bielefeld University, Bielefeld, Germany

## Abstract

**Background:**

In the age of ‘infodemic,’ children and young people encounter health-related information in digital environments at an early age. They should therefore learn how to deal with these in a health-competent way while still at school. Above all, it is relevant that they learn how to distinguish good health information from false. This can be achieved by promoting health literacy. The intervention “Toolbox” combines areas of media education in schools and health literacy to be implemented in schools and curricula in the long term.

**Methods:**

The intervention was designed to address existing health topics in the classroom while strengthening digital health literacy in grades 7 to 10. The Toolbox was piloted in three school classes in Germany and the feedback provided by teachers and students was implemented in a new version of the intervention.

**Results:**

The basic understanding of health literacy in the Toolbox is finding, understanding, evaluating and using digital health information. These steps are followed through using quality criteria for good health information. Students learn that they need to find out the type of information, analyze the author, investigate the media provider, and also the sources given. If they are unsure, they can conduct an adapted questionnaire regarding identify misinformation and disinformation. In addition, the students and teachers receive working materials for group work.

**Conclusions:**

Recognizing disinformation is a relevant skill already for children and adolescents. The intervention should make it easier for students to recognize good health information. For teachers it should be made easier to address health literacy in school without additional effort.

